# Frontal and Axial Evaluation of Craniofacial Morphology in Repaired Unilateral Cleft Lip and Palate Patients Utilizing Cone Beam Computed Tomography; An Observational Study

**DOI:** 10.3390/ijerph17217786

**Published:** 2020-10-24

**Authors:** Anuraj Singh Kochhar, Maninder Singh Sidhu, Mona Prabhakar, Ritasha Bhasin, Gulsheen Kaur Kochhar, Himanshu Dadlani, Gianrico Spagnuolo

**Affiliations:** 1 Former Orthodontist Max Hospital Gurgaon, Haryana 122001, India; anuraj_kochhar@yahoo.co.in; 2 Department of Orthodontics, Faculty of Dental Sciences, SGT University Gurugram, Haryana 122006, India; deanresearch@sgtuniversity.org (M.S.S.); mona.prabhakar@sgtuniversity.org (M.P.); 3 Faculty of Dentistry, University of Toronto, Toronto, ON M5G1G6, Canada; ritasha.bhasin@mail.utoronto.ca; 4 Department of Pediatric & Preventive Dentistry, National Dental College & Hospital, Punjab 140507, India; gulsheenuppal@gmail.com; 5 Department of Periodontology, Kalka Dental College & Hospital, Meerut 250006, India; himdent@hotmail.com; 6 Department of Neurosciences, Reproductive and Odontostomatological Sciences, University of Naples Federico II, 80131 Naples, Italy

**Keywords:** cleft, morphology, unilateral cleft lip and palate, CBCT, arch collapse, asymmetry

## Abstract

The current study was conducted to assess the extent of maxillary arch collapse on the cleft vis-a-vis non-cleft sides in the same individual presenting withunilateral cleft lip and palate (UCLP), using cone-beam computed tomography (CBCT). Thirty-one children (eighteen boys andthirteen girls) with surgically repaired UCLP, who met the inclusion criteria, were selected. Following the acquisition of CBCT scans, fourteen bilateral landmarks were selected. The distance of the bilateral landmark was calculated from the midsagittal plane on the cleft and non-cleft sides for both frontal and axial views. Tracings were done;the data obtained was subjected to statistical analysis;and intra-observer variability was checked with intraclass correlation coefficient (ICC) and two-way ANOVA. Subsequently, the measurements were subjected to paired *t*-tests at the 95% level of significance with Bonferroni correction. A significant reduction of pyriforme and an alveolar crest above the maxillary 1st molar were discerned in frontal analysis on the cleft side. In the axial view, the zygomatic arch, malar, porion and alveolar crest at the molar region were non-significant, but the alveolar crest at the premolar region (*p* < 0.004)) was significantly decreased. In the frontal analysis, pyriforme and the alveolar crest above the maxillary 1st molar, and, in the axial view, premolar widths, showed significant reduction when comparing the cleft vis-a-vis non-cleft sides.

## 1. Introduction

A vital determinant while assessing cleft lip and/or palate (CL ± *p*) treatment qualitatively and quantitatively is midfacial growth, which plays a pivotal role in prognosis. The greatest cause for hypoplastic maxilla in these patients is probably iatrogenic ramifications induced by unsatisfactory surgical outcomes, as untreated cleft patients often show usual growth potential [1]. Hence, cognizance of the pre-operative anatomy of the cleft and neighboring regions is imperative for quintessential treatment planning [2]. Moreover, for proper therapeutic decision-making, it is exigent to have proficiency of the effect of CL ± *p* on maxillary and mandibular growth [3]. Almost 3/4 of CL ± *p* patients, have a cleft alveolus in addition, which may lead to anatomic and dental challenges, including impacted teeth, supernumerary teeth, crowding, atendency toward a Class III malocclusion, and crossbites, which often necessitate orthodontic intervention [4,5].

Various methods for morphological assessment have been utilized in the past, such as direct physical measurements, appraisal of standardized clinical photographs [6], and dental casts [7,8]. Owing to the distortion of reference points and planes in cleft lip and palate (CLP) patients, the detection of anatomical variations is complex and arduous. However, three-dimensional computed tomography (3D-CT) has been found to be beneficial in craniofacial asymmetry diagnosis because, in 3D-CT, regional anatomy is shown in a series of cross-sectional axial images unobstructed by other anatomic features [9].

In lieu of the advantages of cone-beam computed tomography (CBCT), such as reduced scanning time and diminished radiation exposure compared to conventional CT, CBCT is used to identify root resorption caused by impacted or supernumerary teeth, craniofacial clefts and syndromes, and temporomandibular joints, as well as asymmetry assessment [3,10].

There is a deluge of studies utilizing CT or cephalograms to compare transverse dimensions within and with non-cleft individuals [8,9]. Studies found narrower maxillary alveolar and intermolar widths compared to non-cleft individuals in unilateral cleft lip and palate patients [5,11]. Moreover, Buyuk et al. revealed that interorbital, maxillary skeletal widths were reduced in patients with unilateral cleft lip and palate (UCLP) [5]. However, contrasting studies by Abuhijleh et al. found no such difference and reported a significant increase in the nasal cavity width in patients with UCLP [12]. Suri et al. in their interesting study using CT scans in a custom made cephalostat, concluded that the asymmetry was in the dento-alveolar area near the cleft and the nasal chamber and not in the deeper regions [9].

Requisite for comprehensive treatment in UCLP patients is not only the treatment of sagittal discrepancy but also of the transverse dimension because, in the end, a symmetrical morphological outcome is desired [8]. However, there is a scarcity of data available on using CBCT for asymmetry evaluation of the cleft and non-cleft sides in the same patient. Therefore, the aim of this observational study was to quantitatively assess the extent of maxillary arch collapse on the cleft vis-a-vis the non-cleft side using CBCT.

## 2. Materials and Methods 

### 2.1. Subjects

The study sample was calculated at power 80%, alpha level 0.5, and anticipation of large effect size (0.8). Based on this, the power analysis showed that at least twenty-one patients were required [13]. Fifty-four North Indian children in the age group of ten to fourteen years with previously surgically repaired complete CL ± *p* anomalies were examined. Of these, those having no secondary alveolar bone grafts done with primary CLP repair performed before eighteen months of age, having had the same primary surgical techniques (modified Millard procedure) performed by different doctors, having no prior orthodontic/orthopedic appliance intervention, and having been recommended for comprehensive orthodontic treatment in the most recent assessment were selected. Patients with any craniofacialanomalies, syndromes or mental retardation were excluded. Thirty-one children (Table 1) fulfilling the inclusion criteria were selected and designated as samples. Informed consent and willingness to participate was obtained.

### 2.2. Measurements and Data Acquisition

**Figure 1:**
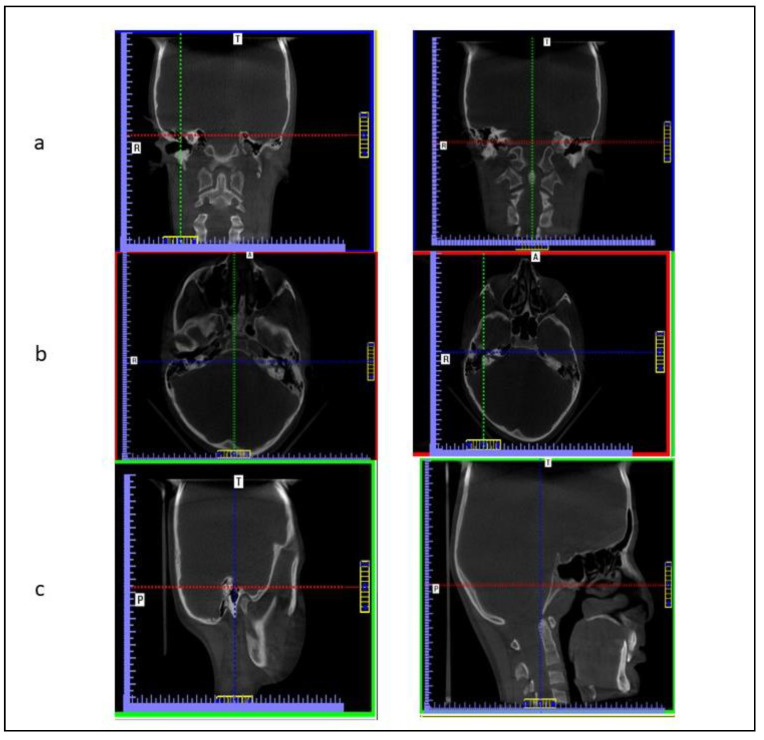
(**a**) Volume rotated mediolaterally until the transporionic line of the data became horizontal. (**b**) Volume rotated until the midsagittal plane of the data oriented vertically. (**c**) Sagittal view for which the Frankfort plane of the data was oriented horizontally. T is Transverse, A is Axial, R is Right side, P is Posterior

Following the acquisition of CBCT scans that were performed as an investigation for the comprehensive evaluation and treatment of cleft lip and palate in the patients using an i-CAT next-generation machine (Imaging Sciences International, Hatfield, PA (field of view: 17 × 22 cm), the data was saved in DICOM (version 1.7)) format with an isometric voxel size of 0.25 mm and then reoriented utilizing InVivoDental 5.0 (Anatomage, anatomy imaging software San Jose, CA, USA). For standardization, the volumes were reoriented (Figure 1). Fourteen bilateral landmarks were used, which are listed in Table 2. The landmarks were chosen by referring to the previous published studies, along with the landmarks that are inline or near the cleft portion of the maxilla and frequently present morphological and anatomical variation [5,9,10].

Each of the images was imported into the nemoceph NX software (Visiodent, Saint-Denis, France) for linear measurements. The distance of the bilateral landmark was calculated from the midsagittal plane on the cleft and non-cleft sides for bothfrontal and axial views. The data obtained weresubjected to statistical analysis.

### 2.3. Statistical Analysis

To check intra-observer reliability, point identification was done five times by the same operator, with a gap of ten days. The intraclass correlation coefficient (ICC) for intra-observer reliability with2-way ANOVA with 2 degrees of freedom was plotted for all paired measurements. A high level of reproducibility of the method of analysis was validated for each landmark with ICC; this was more than 0.90, which was considered a reasonably good measure for agreement. Hence, all the measurements were subjected to paired *t*-tests at the 95% level of significance. Bonferroni correction was done as independent statistical tests were being performed simultaneously on a single data set. Since fourteen landmarks were evaluated, the cut-off value for significance was 0.05/14 = 0.004.

### 2.4. Ethical Considerations

Following the approval of the study bythe institutional ethical committee of SGT Dental College, Hospital and Research Institute, Budhera, Gurgaon, India (SGTDC/PPL/Com./E.C./14 Aug 2010), this study was conducted at the Department of Orthodontics & Dentofacial Orthopedics, Faculty of Dentistry, SGT University, India from March 2011 to May 2013. The research objectives were explained to the patients’ caregivers, and participation was voluntary. It was ensured that each individual understood that the data obtained were used for research only and that all records were confidential.

## 3. Results

Intra-examiner reliability tests showed that the repeatability of the measurements was excellent (0.978). The measurements of the arch collapses of thirty-one unilateral patients and their results for the paired *t*-test at the 95% level of significance are shown in Table 3. In the frontal view, though there was a difference between the cleft and non-cleft sides for the zygomaticofrontal suture, infraorbital margin, malare, lower 1st molar, mental foramen, antegonial notch, and gonion, when measured with respect to the midsagittal plane, the values were not statistically significant. Significant reduction in the transverse position of pyriforme was noted on the cleft side (cleft side, 7.77 ± 2.261 mm; non-cleft side, 5.3 ± 1.504 mm; (*p* **<** 0.004). Asignificant reduction was seen in the alveolar crest above themaxillary 1st molar on the cleft side (cleft side, 11.49 ± 2.716 mm; non-cleft side, 13.65 ± 3.685 mm (*p* ˂ 0.004). 

In the axial view, the points on the zygomatic arch, malar, porion and alveolar crest over the molar region were non-significant, whereas the alveolar crest at the premolar region on the cleft and non-cleft sides showed significant reduction (cleft side, 7.55 ± 3.875 mm; non-cleft side,9.06 ± 3.931 mm) (*p* < 0.004).

## 4. Discussion

Apparent facial asymmetry is perceivable in children born with facial clefts owing to congenital deformity. Quantitative assessment of these asymmetries is arduous with two-dimensional photography and radiographic studies [5]. Nevertheless, emerging advancements in three-dimensional surface imaging (3D stereophotogrammetry) allow quantitative evaluation of topographic regional anatomy. Conventionally, in conjunction with dental casts, posterior-anterior (PA) cephalometric radiographic images have been used to assess maxillofacial asymmetry [7,14]. Magnification, distortion, and superimposition of craniofacial anatomy are a few of the drawbacks of PA cephalometries. To overcome these shortcomings, CBCT, which provides unobstructed views of bilateral landmarks, 3D reconstruction, and numerous other advantages, has been deployed as the radiographic instrument in this study.

A bibliographic search using Medline, PubMed, Scopus, and Google Scholar with the keywords “CBCT”, “Unilateral cleft”, “UCLP”, and “asymmetry” showed a paucity of erudition on CBCT images of the transverse craniofacial morphology of UCLP in the literature. Therefore, the present study was undertaken. 

### 4.1. Frontal View Measurements for Asymmetry Evaluation 

In alandmark study on unilateral cleft lip and palate asymmetry, Mølsted and Dahl [15] found insignificant findings in the region of the lateral orbital margin but feebly significant findings on the medial orbital margin. These findings were in accordance with the present study, where the zygomaticofrontal sutureand the infraorbital foramen revealed statistically insignificant differences. Moreover, Gorucu-Coskuner et al. [8], Duffy et al. (2000) [16], Hood et al. [17], and Suri et al. [9] also suggested insignificant craniofacial asymmetry of the deeper midfacial regions whereas Buyuk et al. [5] concluded a significantly reduced interorbital width in cleft patients. Moreover, Harikrishnan et al. [10] also divulged that the asymmetry in UCLP is not limited to the maxilla, instead emanating to the orbital and zygomatic regions. In consensus, Patel et al. [18] further advocated this, as they found significant differences that not only included the nasal pyriforme region but also the zygoma and mandible.

In the present study, a significant reduction in the transverse position of pyriforme was observed on the cleft side (*p* < 0.004). This finding was in unanimity with Mølsted and Dahl [15] and Mølsted et al. [19], who also compared the cleft and non-cleft sides and discerned decreased nasal cavity dimensions. A considerably significant sagittal depression of the alar base (pyriforme) was detected by Suri et al. [9]. Though the total nasal width was significant in the unilateral complete CLP group, there were no significant differences between the control and unilateral complete CLP groups in zygomatic arch width, maxillary, and condylar width, according to Abuhijleh et al. [12]. Hence, the operator must be cognizant of the possible sagittal as well as transverse asymmetry of the bony alar base, upper midface (zygoma), and basal dentoalveolar complex for treatment planning utilizing alveolar bone grafts and orthognathic surgery [9,18,20].

A significant reduction was witnessed in the alveolar crests above the maxillary first molar on the cleft side (*p* < 0.004) in the frontal view in the current study. The processusalveolaris, the most prominent point on the alveolar process, presented significant differences between unilateral CLP andcleft lip patients [15,19]. Similar results were obtained by Suri et al. and Buyuk et al., with significant findings when the maxillo-alveolar prominence was evaluated on the cleft vis-a-vis the non-cleft side [5,9].

However, when comparing the measurements of the alveolar crests at the mandibular molar region, the results were insignificant. It was contemplated by Shrestha et al. that, in the mandible, unceasing remodelling of the cortical bone, remineralization, and increased bone density are observed when subjected to heavy loads, such as forces of mastication, irrespective of the presence of an alveolar and/or palatal cleft [3,21].

In the present study, theantegonial notch and gonion, measured with respect to the midsagittal plane, were not statistically significant. Mølsted et al. [19] also presented similar findings in a multicenter examination. The Gonion–Gnation angle is a very useful diagnostic parameter to consider before starting an orthodontic treatment because it evaluates the facial pattern of a subject and reflects the variability of the mandibular plane in relation to the anterior cranial base [22]. Kyrkanides and Richter [23] inferred that antegonial notching, though statistically insignificant between cleft and non-cleft individuals, provided a preliminary manifestation of developing mandibular and lower facial asymmetry. Likewise, Buyuk et al. andAbuhijleh et al. concluded that antegonial notching in individuals with UCLP was statistically insignificant [5,12]. However, when evaluating different growth periods, significant differences were observed at different growth periods according to Abuhijleh et al. [12].

According to Veli et al. [24], UCLP patients have symmetrical mandibles when comparing cleft and non-cleft sides, which was in accordance with our study, where we found non-significant measurements in mandibles. However, the Gonion-Gnathion angle was the shortest in the UCLP and bilateral cleft lip and palate (BCLP) groups compared with the control group in a study by Srestha et al. [3]. Abuhijleh et al. [12] illustrated that a short, retrusive, and posteriorly rotated mandible could be due to the short cranial base and mandibular adaptation to an underdeveloped maxilla.

### 4.2. Axial View Measurements for Asymmetry Evaluation

In the axial view, when the cleft and non-cleft sides were juxtaposed, the deeper structures presented with non-statistical differences in the present study; this was in accordance with Suri et al. [9]. However, it was in contrast with the findings of Harikrishnan et al. [10] and Patel [18].

Points on the malare and the zygomatic arch showed non-significant findings in the axial view. The point on the porion was also non-significant in our study and was in conjugation with Suri et al. [9], who found analogous results in both sagittal and transverse sections. Choi et al. [25] gauged the positional symmetry of the porion and the external auditory meatus in facial asymmetry patients and suggested that the porion tends to have symmetrical vertical locations in symmetrical and asymmetrical subjects.

In the axial view, the alveolar crest at the premolar regions showed significant findings in the present study. Disthaporn et al. [7] concluded that the arch collapse was pronounced at the deciduous canines and gradually decreased in the posterior portion of the arch with inconsequential measurement, although statistically significant at the maxillary first permanent molar region. This was in conjugation with the present study, with the canine premolar region being significant and the molar region being insignificant. Suri et al. [9] and Harikrishnan et al. [10] also found a congruent pattern of arch constriction. Additionally in agreement, but not statistically significant, were the results of Gopinath et al., who found a narrower maxillary arch width in the intercanines and the inter first premolar regions in the UCLP group [26]. Generali et al.(2017) found no significant reduction of the intermolar widths in the mixed dentition phase; however, the differences were significant only for the intercanine widths between the UCLP and non-cleft groups. They postulated the more substantial hypoplasia, with constriction of the anterior compared to the posterior portion of the maxilla, to be responsible for the less pronounced narrowing of the intermolar widths in the maxillary arch [27]. 

Mølsted and Dahl [19] pointed out that maxillary width decreased after the primary operation. The current study’s results corroborate the findings of some others, whonoted the greatest arch constriction at the unilateral cleft side [28]. The clinical ramification of arch collapse is that, during the introductory phase of orthodontics preceding alveolar grafting, expansion must be optimized and individualized [7,20]. Al-Gunaid [29] concluded that the maxillary arch forms in UCLP patients might play a preeminent role in the stabilization of the maxillary dental arch widths following orthodontic treatment and diminish the propensity to relapse. Reconnoitring definite points of constriction aids in the selection of the most relevant expanders, in gaining an adequate amount of expansion, and in decreasing post treatment relapse.

### 4.3. Limitations

The primary limitation can be attributed to non-inclusion of bilateral cleft lip and palate patients, who also constitute a large part of patients with cleft lip and palate. Although, in the present study, intraobserver variation was found to be good with ICC (0.9), identification of landmarks in cases of cleft lip and palate are arduous, and not all observers are able to localize the points in a similar manner.

## 5. Conclusions

In the frontal analysis, the area encompassing the cleft region showed significant reduction in pyriforme and the alveolar crest over the maxillary first molarwhen compared to the non-cleft side. In the axial view, when comparing the cleft vis-a-vis the non-cleft sides, the premolar widths were significantly reduced.

## Figures and Tables

**Table 1:** Sample characteristics.

Gender	Boys (*n* = 18)			Girls (*n* = 13)			Total Sample (*n* = 31)		
	Mean	SD	Range	Mean	SD	Range	Mean	SD	Range
Age (years.)	12.035	0.690	10–14	12.13	0.724	10–14	12.09	0.698	10–14

**Table 2:** Landmark definitions for asymmetry evaluation.

Frontal View		
1.	Zygomaticofrontal suture (ZFS)	Intersection of zygomatic frontal suture and lateral orbital margin
2.	Infraorbital foramen (IOF)	Medial point of infraorbital foramen
3.	AMC	Alveolar crest over maxillary first molar (AMC)
4.	Ante gonial notch (AGN)	Deepest point of ante gonial notch of mandible
5.	Gonion (Go)	Point along angle of mandible midway between lower border of mandible and posterior ascending ramus
6.	Pyriforme (Py)	The most lateral aspect of the piriform aperture
7.	Malare(Mf)	Most prominent anterolateral point on the zygomatic bone that lends the malar prominence to the face
8.	Lower first molar (LFM)	Crest of bone buccal to mandibular first molar
9.	Mental foramen (MF)	Foramen located on anterior surface of the mandible
**Axial View**		
10.	Zygomatic arch (ZA)	A point at the most lateral surface of the zygomatic arch near thezygomatic-maxillary suture
11.	CMP	Crest of bone palatal of maxillary 1st premolar region (CMP)
12.	CMM	Crest of bone palatal of maxillary 1st molar region (CMM)
13.	Malare (Ma)	Most prominent antero-lateral point on the zygomatic bone that lends the malar prominence to the face
14.	Porion (Po)	Centre of the tangent connecting the anterior and posterior bony limits of the lateral extent of the external auditory meatus.

**Table 3:** Comparison of the values between the cleft and non-cleft sides.

S.No.	Variables	N	Cleft		Non-Cleft		*t*-Value	*p*-Value
			Mean	SD	Mean	SD		
**Frontal View**								
1	Zygomatico frontal suture	31	24.79	5.62	24.53	5.713	1.667	0.116
2	Infraorbital foramen	31	13.66	3.079	13.75	2.888	0.304	0.765
3	Alveolar crest over maxillary molar	31	11.49	2.716	13.65	3.685	5.202	0.001 *
4	Antegonial notch	31	20.02	6.137	19.75	5.862	0.994	0.336
5	Gonion	31	24.21	7.488	24.46	7.716	0.573	0.575
6	Pyriforme	31	7.77	2.261	5.3	1.504	6.743	0.001 *
7	Malare	31	10.85	1.946	11.24	2.034	1.473	0.34
8.	Alveolar crest on mandibular first molar	31	10.04	1.89	10.54	2.003	1.365	0.812
9.	Mental foramen	31	8.34	2.708	8.57	2.56	1.237	0.723
**Axial View**								
10.	Point of zygomatic arch	31	29.85	8.152	30.41	8.027	1.059	0.306
11.	Alveolar crest at maxillary pre molar region	31	7.55	3.875	9.06	3.931	3.49	0.003*
12.	Alveolar crest at maxillary molar region	31	8.53	2.102	10.07	2.384	3.11	0.007
13.	Malare	31	9.48	1.87	9.65	1.59	1.028	0.401
14.	Porion	31	25.67	5.134	26.014	5.027	1.184	0.516

* Significant (*p* < 0.004).
